# Use of antipsychotic and antidepressant within the Psychiatric Disease Centre, Regional Health Service of Ferrara

**DOI:** 10.1186/1472-6904-11-21

**Published:** 2011-12-20

**Authors:** Stefano Bianchi, Erica Bianchini, Paola Scanavacca

**Affiliations:** 1 Department of Pharmacy, University Hospital of Ferrara, Corso Giovecca 203, 44123 Ferrara (Italy

## Abstract

**Background:**

This study aimed at describing the type and dosage of psychopharmaceuticals dispensed to patients with psychiatric disorders and to assess the percentage of patients treated with antipsychotics and antidepressants, the associated therapies, treatment adherence, and dosages used in individuals registered at the Psychiatric Disease Center (PDC), Regional Health Service of Ferrara.

**Methods:**

The analysis focused on therapeutic programmes presented to the Department of Pharmacy of the University Hospital of Ferrara of 892 patients treated by the PDC (catchment area of 134605 inhabitants). All diagnoses were made according to International Classification of Diseases (ICD-9). The analysis focused on prescriptions from September 2007 to June 2009. Data on adherence to prescribed therapy have were processed by analysis of variance.

**Results:**

Among the patients 63% were treated with antipsychotics and 40% with antidepressants. Among patients receiving antipsychotics 92% used second-generation antipsychotics (SGAs) whereas the remaining 8% used first generation antipsychotics (FGAs). Antipsychotic doses were lower than Daily Defined Dose (DDDs), and SGAs were often given with anticholinergics to decrease side effects. Mean adherence to antipsychotic therapy was 64%. Among antidepressants, selective serotonin reuptake inhibitors (SSRIs) were the most often prescribed, 55%. Dosages of these were within the limits indicated by the technical datasheet but higher than DDDs. Only 26% of patients underwent monotherapy. In antidepressants polytherapy, medication was associated with another antidepressant, 6% or with an antipsychotic, 51%. Mean adherence to the antidepressant therapy was 64%.

**Conclusions:**

Patients treated with antipsychotics tend to use doses lower than DDDs. The opposite tendency was noted in patients treated with antidepressants. Only a small percentage of patients (14%) modified their neuroleptic therapy by increasing the dosage. On the contrary, patients treated with antidepressants mainly tended to reduce the doses of their drugs. This study highlights the tendency to follow combination therapies, prescribing SGAs together with anticholinergics in order to minimize extrapyramidal side effects or by combining two antidepressants. The study showed low adherence for both pharmaceutical therapies, which is typical in the setting of the analyzed diseases.

## Background

Psychiatric disorders including different forms of depression and psychosis are highly debilitating conditions that generate strong discomfort in affected patients and a heavy burden on society as a whole; drugs indicated for these are expensive and treatment for these patients costs more than the average cost of care for other common diseases such as diabetes and hypertension.

Several studies [1,2] demonstrated good efficacy of antipsychotics and antidepressants in alleviating mental disorders such as schizophrenia, depression, and bipolar disorder. However, the efficacy of controlling the symptoms of these disorders clearly depends on patient adherence to treatments, which is typically unsatisfactory [3].

Literature reports [4,5] show significant differences between prescriptions in the clinical setting of psychiatric disorders and recommendations in official guidelines. In particular, studies on prescription of drugs have highlighted the frequent co-prescription of two or more medications [6-10], including anticholinergics [11,12], antidepressants, and antiepileptic drugs. Moreover, further studies [13] have shown that a high percentage of users receive drugs that affect the CNS above defined therapeutic ranges [14,15].

In this study, we looked at the patterns of antipsychotic and antidepressant use within the Psychiatric Disease Centre (PDC), Regional Health Service of Ferrara, Italy.

## Method

### Study design

This was a descriptive, retrospective single-centre study focusing on drugs prescribed by the psychiatrists of the PDC, with a catchment area of 134605 inhabitants, and dispensed by the Department of Pharmacy of the University Hospital of Ferrara.

The study mainly focused on patients treated at the PDC with antipsychotics (Anatomical Therapeutic Chemical [ATC] N05) and antidepressants (ATC N06), even when associated with CNS-active drugs (ATC N). ATC is a classification system in which drugs are divided into different groups depending on the organ and their chemical, pharmacological, and therapeutic properties. Drug prescriptions including CNS-inactive drugs were not taken into account.

The observation period was 26 months from March 2007 to 31 May 2009. The number of patients evaluated during the evaluation period was 911.

Since the diagnoses for 21% of patients were incomplete, it was not possible to obtain definitive diagnoses for all patients considered in the study. Furthermore, 19 patients (2%) were ineligible for the study because they did not take drugs specified in the inclusion criteria mentioned above. Therefore, the total number of patients eligible for this study was 892 (98%). Drugs prescribed to patients treated by the PDC were all dispensed by the hospital pharmacy.

### Subjects

This study was approved by the Ethics Committee of Ferrara on 28/07/2011 and conducted by applying the legislative decree of June 24, 2003, no. 211 of Good Clinical Practice in clinical trials of medicines for clinical use. The study was conducted in accordance with the Declaration of Helsinki.

### Efficacy and safety assessment

This study aimed to assess the use of antipsychotic agents (ATC N05) and antidepressants (ATC N06) dispensed to patients treated at the PDC, Regional Health Service of Ferrara. More specifically, it aimed to assess:

• clinical characteristics of patients;

• type of drugs prescribed and their dosage;

• doses used compared with those indicated by drug technical datasheets (Summary Product Characteristics; SPC) and the Daily Defined Dose (DDD) [16];

• potential modifications to therapy (change of active drugs and their doses or addition of other active drugs);

• percent patients treated with polytherapy and analysis of the most frequent combinations;

• patient adherence to prescribed therapies, calculated as percentage drugs consumed versus quantity prescribed.

### Description of instruments

Patients treated at the PDC obtained drugs prescribed by psychiatrists from the Department of Pharmacy of the University Hospital of Ferrara. The pharmacist completed the dispensation database including the following information: patient data (name, date of birth); prescribing specialist; diagnosis (coded as ICD-9), prescription (limitation period; prescribed medication; dosage; pharmaceutical form; routes of administration; active substance or drug); and amount of medication dispensed calculated on the basis of previous drug dispensations.

From that database, we analyzed the following data: overview of patient data, pathology according to ICD-9, prescribing centre, number of tablets/capsules/vials distributed, duration of prescription, daily dosage, quantity consumed. All variations made by doctors on patients' prescriptions concerning routes and time of administration were also taken into account.

We calculated the minimum, average, and maximum doses of the psychopharmaceuticals used in therapies and compared them with the DDDs as well as the minimum and maximum dosages of typical and atypical drugs listed in the SPCs.

Being the only daily average dosage internationally recognized, the DDD value was chosen as a parameter to compare daily dosages of therapy. Moreover, the dosage corresponding to the maximum frequency used in therapy was calculated for each drug.

Patient level of adherence to prescribed therapies was also assessed, calculated as the ratio between the amount of drug they actually withdrew to that prescribed by the doctor.

### Statistics

Data on adherence to prescribed therapy were assessed by analysis of variance (ANOVA). Adherence was reported as the mean and its confidence interval 95% to asses whether is a difference in adherence of the various molecules. For statistical analysis we used SPSS 18 software.

A t-test was performed on the average adherence of patients treated with FGAs compared with SGAs.

## Results

### Patients

A total of 892 patients were eligible for the study (M/F, 362/530); their clinical characteristics are shown in Table 1.

**Table 1:** Features of patients eligible for the study

Parameter	n	%
Men	362	40.58
Women	530	59.42
**Age group**		
10-20	8	0.93
21-30	50	5.61
31-40	124	13.90
41-50	190	21.30
51-60	167	18.72
61-70	154	17.26
71-80	133	14.91
81-90	61	6.84
91-100	5	0.56
Mean age, years	56 ± 10.09	

SD, standard deviation.

Mood disorders (especially major depressive disorder and bipolar disorder) and schizophrenia were the most frequently diagnosed conditions, accounting for 36% and 35%, respectively, for a combined total of 72% of all diagnoses (Table 2).

**Table 2:** Diagnosis reported in therapeutic programs and coded with the ICD-9 system

ICD-9	Correspondence	%
**296**	Mood disorders	**36.4**
**295**	Schizophrenia	**35.4**
**298**	Non-organic psychosis	**5.6**
**299**	Pervasive developmental disorders	**4.7**
**297**	Delusional disorders	**4**
**290**	Dementia	**3.9**
**294**	Persistent mental illnesses caused by pathologic conditions	**3**
**292**	Mental disorder caused by substances	**2.4**
**293**	Temporary mental illnesses caused by pathologic conditions	**2**
**300**	Anxiety disorders	**1.42**
**309**	Adaptation reactions	**0.85**
**318.1**	Severe mental retardation	**0.28**
**307.1**	Anorexia nervosa	**0.14**

ICD, International Classification of Diseases.

### Antipsychotics

In all, 63% of patients (564/892) were prescribed antipsychotics; 42% of them (235/564) were men and 58% (329/564) women. Their age range was 15-98 years and most (40% of the total) were in the age range 41-60 years, although a few prescriptions were made to patients aged <20 years (0.5%) and in their 90s (1%). Mean age of the analyzed population was 55 years (SD % ± 17).

### Type of prescribed drugs

In patients on antipsychotics, 520/564 (92%) took second-generation antipsychotics (SGAs) whereas the remaining 8% (44/564) took first-generation antipsychotics (FGAs). The distribution is shown in Table 3.

**Table 3:** Patients treated with antipsychotics and treatment adherence

Drug	n	% patients	Adherence			CI 95%	P-value
			Minimum	Mean	Maximum		
	**SGAs**						

Aripiprazole	43	7.6	7.78	62.07	100	22.6 - 80.1	0.01
Olanzapine	193	34.2	7.78	59.15	100	25.9 - 81.2	0.01
Quetiapine	156	27.7	8.33	66.68	100	27.9 - 88.5	0.01
Risperidone	109	19.3	8.33	59.75	100	29.3 -84.3	0.01
Clozapine	19	3.4	7.8	51.80	100	29.7 - 85.2	0.01

	**FGAs**						

Haloperidol	26	4.6	8.33	63.99	100	17.4 - 59.2	0.01
Clotiapine	8	1.4	16	66.38	100	24.3 - 96.5	0.01
Levomepromazine	6	1.1	33.33	68.06	100	32.8 - 87.9	0.01
Clorpromazine	4	0.7	22.22	69.44	100	20.4 - 80.0	0.01

SGA, second-generation antipsychotics;

FGA, first-generation antipsychotic.

### Drug doses

Mean doses were consistently lower than those indicated by the DDDs. Namely, drugs with prescribed doses slightly lower than the DDDs were: aripiprazole 13.2 mg (DDD, 15 mg); olanzapine 8.6 mg (DDD, 10 mg); risperidone 3.7 mg (DDD, 5 mg); and haloperidol 3.7 mg (DDD, 8 mg) (Table 4). Drugs with prescribed doses much lower than the DDDs were: quetiapine 230.6 mg (DDD, 400 mg); clozapine 179.5 mg (DDD, 300 mg); clotiapine 38.6 mg (DDD, 80 mg); levomepromazine 25 mg (DDD, 300 mg); chlorpromazine 62.5 mg (DDD, 300 mg) (Table 4).

**Table 4:** Antipsychotic dosages in milligrams used in therapy

Drug	DDD	Dosage			SPC	Most commonly prescribed dosage, mg	% Patients with add-on anticholinergic
					
		Minimum	Mean	Maximum			
**SGAs**							

Aripiprazole	15	2.5	13.16	30	10-30	10	2.3
Olanzapine	10	1.3	8.6	20	5-20	5	6.7
Quetiapine	400	12.5	230.6	1200	200-800	200	2.6
Risperidone	5	0.5	3.7	9	2-16	2	11
Clozapine	300	25	179.5	450	200-450	100	10.5
**FGAs**							
Haloperidol	8	1	3.71	8	60	2	27
Clotiapine	80	14	38.6	100	100-120	50	87.6
Levomepromazine	300	12.5	25	50	75-300	25	0
Chlorpromazine	300	62.5	62.5	100	30-75	50	50.4

Trascrizione fonetica

DDD, Defined Daily Dose;

SPC, Summary of Product Characteristics;

SGA, second-generation antipsychotic;

FGA, first-generation antipsychotics.

Moreover, the following drugs were prescribed at doses lower than those described in the SPCs: clozapine 179.5 mg (SPC, 200-450 mg); haloperidol 3.71 mg (SPC, 60 mg); clotiapine 38.6 mg (SPC, 100-120 mg); and levomepromazine 25 mg (SPC 75-300 mg) (Table 4).

### Variations in therapy

In all, 16% of the analyzed population (95/564 patients) changed their drug dosage during therapy: 44/564 patients (8%) reduced it while 51/564 patients (9%) increased it. More specifically, increases in prescribed doses of the following drugs were noted: quetiapine (24/51), olanzapine (17/51), aripiprazole (4/51), haloperidol (3/51) and risperidone (3/51). Antipsychotics that were dose-reduced were olanzapine (25/44), aripiprazole (7/44), quetiapine (6/44), risperidone (5/44), and haloperidol (1/44).

The originally prescribed drug was switched to other medication in 14% of patients (81/564). As such, the following antipsychotics were switched: olanzapine 0.8%; risperidone 4%; quetiapine 3%; aripiprazole 1%; clotiapine 0.4%; haloperidol 0.4%; and chlorpromazine 0.2%.

### Concomitant medications used with antipsychotics

In all, 427/564 patients (76%) were treated with antipsychotics and other concomitant therapies. Concomitant therapies prescribed with antipsychotics included the following: antidepressants 40% (228/564); antiepileptics 16% (88/564); anticholinergic 9% (48/564); antiepileptic and antidepressant 7% (40/564); anticholinergic and antidepressant 2% (12/564); anticholinergic and antiepileptic 1% (8/564); and anticholinergic, antidepressant, and antiepileptic 0.5% (3/564).

Among patients taking an antipsychotic with an antidepressant, 83% were on SGAs. Moreover, SGAs were frequently given with anticholinergics (73%).

### Adherence to therapy

Patient adherence to therapy is shown in Table 3; this parameter was calculated ranging from a minimum of 60% for risperidone to a maximum of 69% for chlorpromazine. Mean adherence to treatments was 64% (SD% ± 28).

We used the statistical t-test to evaluate and compare the average value of first-generation antipsychotic adherence to that of the second generation. For FGAs, the adherence mean was 66.97 (SD% ± 2.35), while for SGAs it was 59.89 (SD% ± 5.37). This statistical testing (95% confidence interval) resulted in a significant p-value equal to.038, showing a difference of treatment adherence among patients receiving the two categories of antipsychotics, slightly higher for patients using FGAs compared with patients treated with SGAs.

### Antidepressants

Among patients treated with psychopharmaceuticals, 40% (361/892) took antidepressants, among whom 63% (228/361) were women and 37% (133/361) men. Their mean age was 55 years (SD% ± 9). The most frequently observed age group was 41-60 years (43%). However, a small number of prescriptions to patients aged 21-30 years (5%) and 81-90 years (4%) were also made.

### Type of prescribed drugs

Prescribed antidepressants are shown in Table 5. The most prescribed antidepressant was venlafaxine (24%) whereas the most prescribed drug category was selective serotonin reuptake inhibitors (SSRIs) at 55%.

**Table 5:** Patients treated with antidepressants and treatment adherence

Drug	n	% patients	Adherence			CI 95%	P-value
			Minimum	Mean	Maximum		
Paroxetine	75	20.83	7.78	57.7	100	23.8 - 76.5	0.03
Citalopram	83	23.06	7.78	77.2	100	26.0 - 83.6	0.03
Venlafaxine	87	24.17	8.33	61.65	100	24.1 - 81.2	0.03
Sertraline	29	8.06	8.33	62.80	100	23.7 - 85.3	0.03
Mirtazapine	32	8.89	59.14	59.14	100	25.8 - 82.2	0.03
Trazodone	25	6.94	11.11	75.94	100	24.5 - 83.5	0.03
Fluoxetine	10	2.78	13.33	52.18	93.33	18.9 - 73.1	0.03
Amitriptiline	4	1.11	35.71	43.42	50	37.2 - 49.5	0.03
Clomipramine	6	1.67	14.81	67.72	100	23.1 - 84.3	0.03
Reboxetine	10	2.78	33.33	81.07	100	38.6 - 92.5	0.03
**Total**	**361**						

### Drug doses

Minimum, mean, and maximum dosages of antidepressants and their comparison versus the DDDs and the SPCs are displayed in Table 6. The following antidepressants were given at higher doses versus the DDD: citalopram 44.3 mg (DDD, 20 mg); mirtazapine 44.2 mg (DDD, 30 mg); paroxetine 29.5 mg (DDD, 20 mg); sertraline 73.8 mg (DDD, 50 mg); venlafaxine 129.2 mg (DDD, 100 mg).

**Table 6:** Antidepressants dosages in milligrams used in therapy

Drug	DDD	Dosage			Most commonly prescribed dosage, mg	SPC
				
		Minimum	Mean	Maximum		
Citalopram	20	10	44.25	60	20	20-60
Mirtazapine	30	15	44.23	45	30	15-45
Paroxetine	20	5	29.54	60	40	20-60
Sertraline	50	30	73.82	180	100	50-200
Venlafaxine	100	37.5	129.2	300	150	75-375

DDD, Defined Daily Dose;

SPC, Summary of Product Characteristics.

### Variations in therapy

In all, 52 of 361 patients (14%) modified the dose of their therapy: 56% (29/52) of patients reduced it, while the remaining 44% (23/52) increased it.

The following drugs were dose-decreased during the observation period: 19/361 (5%) cases for venlafaxine; 3/361 (0.8%) each for citalopram and paroxetine; 2/361 (0.6%) for amitriptyline; and 1/361 (0.3%) each for sertraline and clomipramine.

On the other hand, patients who increased their drug doses were: 10/361 (3%) for citalopram; 4/361 (1%) for venlafaxine; 3/361 (0.8%) for paroxetine; 2/361 (0.6%) for sertraline; 1/361 (0.3%) each for mirtazapine, fluoxetine, trazodone, and clomipramine.

In all, 6% of patients (22/361) switched their originally prescribed medication to other drugs. More specifically, the following medications were switched to other drugs during the observation period: citalopram 23%; mirtazapine 18%, sertraline and venlafaxine both 14%; trazodone, reboxetine, and fluoxetine all 9%; and paroxetine 5%.

### Concomitant therapies with antidepressants

Only 26% of patients took antidepressants as monotherapy. Drugs taken in addition to antidepressants were: another antidepressant 6% (22/361); antipsychotic 51% (184/361); antipsychotic and antiepileptic 9% (32/361); anticholinergic 0.5% (2/361); antiepileptic 4% (13/361); two antipsychotics 3% (11/361); antipsychotic and anticholinergic 0.3% (1/361); and antipsychotic, anticholinergic, and antiepileptic 0.3% (1/361).

### Mean adherence to therapy

Mean adherence to therapy was: 81% for reboxetine; 77% for citalopram; 76% for trazodone; 59% for mirtazapine; 58% for paroxetine; 63% for sertraline; 62% for venlafaxine; and 52% for fluoxetine. Mean overall adherence to treatments was 64% (SD % ± 34).

## Discussion

### Antipsychotics

Atypical antipsychotics were most frequently prescribed (92%). Among atypical antipsychotics, olanzapine, quetiapine, and risperidone were the most prescribed, whereas among typical antipsychotics haloperidol was by far the most taken.

Mean doses of atypical antipsychotics were within the limits indicated by the SPCs. When compared with the DDDs, although of limited use in such investigations, doses lower than the DDDs and within the limits indicated by the SPCs can mean two different things: on the one hand, low doses can be therapeutically successful, while on the other, their concomitant use allows the reduction of each individual drug dosage as well as of side effects. Moreover, we observed one case of a patient treated with quetiapine at a dosage 1200 mg/day higher than that suggested in the technical datasheet (200-800 mg).

Our analysis of typical neuroleptic daily dispensations showed that prescribed mean dosages were nearly all within the limits indicated by technical datasheets and the DDDs [17] except for two drugs. Once again, this suggests that therapeutic results are achievable at low dosages and that attempts are made to minimize side effects. The only exception here was chlorpromazine, for which maximum doses were higher than those indicated by technical datasheets and the DDDs.

The greatest dosage variations with antipsychotics during the therapy were seen for quetiapine and olanzapine.

The study showed that 14% (81/564) of patients on antipsychotics replaced the initial drug. More specifically, 74/81 (91%) patients initially treated with SGAs were switched to other drugs; only 2/81 (2%) patients replaced the SGAs with FGAs whereas 6% (5/81) of patients switched from FGAs to SGAs. The most often replaced drugs were olanzapine, risperidone, and quetiapine.

The most frequently noted combination therapy was an antipsychotic (mainly SGAs) and an anticholinergic.

SGA dosages used with anticholinergics were consistently lower than the DDDs. Since the risk of extrapyramidal side effects is very low when atypical drugs are taken, the combination use with anticholinergics would be unnecessary. However, the combined use of these drugs was very common and usually higher than FGAs. This therapeutic choice probably aims at further reducing the onset of side effects.

The majority of patients followed prescribed antipsychotic therapy [18] with a low adherence range of 51-66%.

The difference in adherence to treatment (slightly higher among patients treated with FGAs compared with patients treated with SGAs) was statistically significant; however, we recognize the limits of the different types of patients in terms of disease severity. SGAs are currently the first choice of treatment; it often happens that FGAs are reserved to patients as a second choice or where SGAs are contraindicated. Probably for this reason, these patients are the most monitored by the specialists and this could be a reason for greater adherence to therapy for patients treated with FGAs.

In a study by Magliano et al. [6] carried out in 30 Italian mental health services which included 682 patients (M/F 469/213), the data, when compared with our results, revealed some differences. Their study included patients aged 18-60 years (mean age 37 years) and among them, 98% of the subjects used antipsychotics (63% treated with FGAs and 35% with SGAs). In our study, the recruited patients had a wider age range of 15-98 years (and therefore the average age was 55 years old) and only 63% used antipsychotic drugs, with a lower percentage of patients using FGAs (8%) while 92% were treated with SGAs. In our study, the use of combination therapy was more widespread than in the data obtained from the Magliano study, where only 5% of patients were prescribed an antipsychotic and an antidepressant (versus 40% of patients in Ferrara) and 7% were receiving an antipsychotic and an anticholinergic (versus 9% in Ferrara).

### Antidepressants

The most prescribed antidepressant was venlafaxine whereas the most prescribed category of antidepressants, 55% of all antidepressants, was the SSRI class. These data confirm the results highlighted by other studies [19], which showed that SSRIs are the most prescribed antidepressants in Italy. This figure has also been confirmed by other European studies such as those by Finder [19] and Serna [20]. Our analysis shows that doses were almost always within the limits indicated by the technical sheets but consistently above those indicated by the DDDs.

During the therapy, a small proportion (14%) of patients modified their dosage of prescribed drug: 8% of them reduced it while the remaining 6% increased it. The most frequently up-titrated antidepressant was citalopram (19% of the total of patients who increased their therapy) followed by venlafaxine (8%) and paroxetine (6%). On the other hand, it emerged that the prescribed dosage of venlafaxine was usually reduced (in 37% of patients).

Citalopram was apparently not very efficacious based on our finding that it was dose-increased more than other drugs despite having a good adherence.

In most cases, antidepressants were prescribed concomitantly with an antipsychotic or another antidepressant, suggesting quite a high comorbidity of depression and psychosis in patients in this study. The presence of a number of patients taking two antidepressants concomitantly also suggests that depression symptoms are difficult to treat successfully with just a single drug.

## Conclusion

This retrospective study evaluated the prescription profiles of 892 patients treated by the PDC. SGAs were the most commonly prescribed antipsychotic agents. Dosages prescribed were usually within those recommended by the drug manufacturers and international standards. These agents were often used with concomitant therapies, especially with anticholinergics, despite the reduced risk of extrapyramidal side effects. SSRIs were the most commonly prescribed antidepressants, at dosages within the range recommended by suppliers but higher than the DDDs. Antidepressants were mainly prescribed in association with a second antidepressant or an antipsychotic. For both classes of drugs, we observed an average level of patient adherence to their therapies, although there have not been many studies that have investigated adherence to therapy of antipsychotics and antidepressants. Unfortunately, this may represent a limitation to our study because it does not allow comparison of our results to those obtained in other populations.

## List of abbreviations

ATC: Anatomical Therapeutic Chemical; PDC: Psychiatric Disease Centre; SPC: Summary Product Characteristic; DDD: Daily Defined Dose; ICD-9: International Statistical Classification of Diseases and Related Health Problems; SD: standard deviation; SSRI: selective serotonin reuptake inhibitor; SGA: second-generation antipsychotic; FGA: first-generation antipsychotic.

## Competing interests

The authors declare that they have no competing interests.

## Authors' contributions

SB defined the design of this study, establishing the number of patients to be enrolled, the number of prescriptions by therapeutic analysis, and the type of data needed to achieve the defined objectives. SB submitted the study to the Ethics Committee of Ferrara, established the protocols for statistical data analysis, continuously monitored the data obtained, and contributed to the writing and revision of the article. SB defined operating modes, introducing the DDD for comparison of treatments in the dosage given. EB analyzed the requirements of patients and processed the data to obtain patient characteristics, types of drugs prescribed, dosages used in therapy, changes of drug or dosing and possible associations. He participated in drafting and revising the article.

PS evaluated the data obtained and the execution of the study, also participating in the drafting and revision of the article.

All authors read and approved the final manuscript.

## Pre-publication history

The pre-publication history for this paper can be accessed here:

http://www.biomedcentral.com/1472-6904/11/21/prepub
